# A multi-omics systems toxicology framework reveals amino acid–mediated stress adaptation to deltamethrin exposure in a yeast model

**DOI:** 10.1038/s41598-026-66841-2

**Published:** 2026-09-01

**Authors:** Mustafa Yavuz, Hakime Gul Yavuz, Aytac Dursun Oksuzoglu, Ceyhun Bereketoglu, Adil Mardinoglu, Beste Calimlioglu

**Affiliations:** 1 Denizli Food Control Laboratory Directorate, Ministry of Agriculture and Forestry, 20150 Denizli, Turkey; 2 Department of Bioengineering, Marmara University, 34854 Istanbul, Turkey; 3 HealthCentre for Host-Microbiome Interactions, Faculty of Dentistry, Oral & Craniofacial Sciences, King’s College London, London, SE1 9RT UK; 4 Science for Life Laboratory, KTH—Royal Institute of Technology, Stockholm, SE-17165 Sweden; 5 Biotechnology Common Application and Research Center of Excellence (SABIOTEK), 34320 Istanbul, Turkey

**Keywords:** Deltamethrin, *Saccharomyces cerevisiae*, Toxicity, Metabolomics, Transcriptomics, Biochemistry, Computational biology and bioinformatics, Molecular biology

## Abstract

Deltamethrin (DLM) is a widely used synthetic pyrethroid insecticide known for its neurotoxic effects through interactions with voltage-gated sodium channels in insects. Despite its extensive application in agriculture, aquaculture, household settings, and animal husbandry, accumulating evidence indicates that DLM poses significant health risks to non-target organisms, including neurotoxicity, hepatotoxicity, nephrotoxicity, reproductive toxicity, metabolic disorders, and oxidative stress. Elucidating the cellular mode of action of DLM exposure is therefore critical. Previous studies have linked DLM exposure to endoplasmic reticulum (ER) stress and disrupted iron homeostasis; however, the metabolic adaptations underlying these responses remain poorly understood. In this study, we employed an integrated multi-omics approach to investigate the metabolic and transcriptomic responses of Saccharomyces cerevisiae exposed to non-lethal, chronic DLM doses over 30 days. Metabolomic profiling revealed significant alterations in amino acid metabolism, with notable accumulation of glycine and serine, amino acids known to mitigate ER stress. Transcriptomic analysis showed upregulation of iron deprivation response genes (*FRE3*,* SIT1*) and amino acid permease genes (*PUT4*,* AGP1*), indicating enhanced amino acid transport and iron uptake. Our findings demonstrate a coordinated adaptive response in which amino acid trafficking and iron homeostasis act in concert to alleviate DLM-induced ER stress, highlighting conserved stress adaptation mechanisms with potential relevance to human health.

**Supplementary Information:**

The online version contains supplementary material available at https://doi.org/10.1038/s41598-026-66841-2.

## Introduction

The use of insecticides in agricultural practices has steadily increased to prevent food waste and loss caused by insects during preharvest and postharvest periods^1,2^. Naturally derived from *Chrysanthemum cinerariaefolium*, pyrethrins were among the first plant-based insecticides used. However, due to their limited stability and relatively weak insecticidal activity, synthetic pyrethroids were developed as more stable and effective alternatives^3^. Pyrethrins-derived pyrethroids belonging to insecticide group in agriculture primarily exert their effects by targeting the insect nervous system. Following the ban of organophosphates due to their neurotoxic effects in mammals, pyrethroids have become increasingly important in integrated pest management strategies^4^. Given the structural differences among pyrethroids, there are slight differences among them in terms of their mode of actions in insects. While type I pyrethroids alter the charge of voltage-gated sodium channels in insect nerves, type II pyrethroids cause cell membrane depolarization resulting in impeding cellular activities. Deltamethrin (DLM), a type II pyrethroid, has been widely applied to ensure food safety^5,6^. Although DLM is considered less toxic to humans—owing to limited dermal absorption and enzymatic degradation by carboxylesterases—its oral exposure and carboxylesterases inhibition by pyrethroids can lead to severe toxicity in nontarget organisms^3,7,8^;. Recent studies corroborated that by elucidating the link between insecticide exposure and neurological disorders in humans^9,10^. Moreover, pyrethroid exposure has been associated with a range of toxic effects including neurotoxicity, nephrotoxicity, hepatotoxicity, reproductive toxicity, obesity, type 2 diabetes, and oxidative stress^9–11^.

**Fig. 1:**
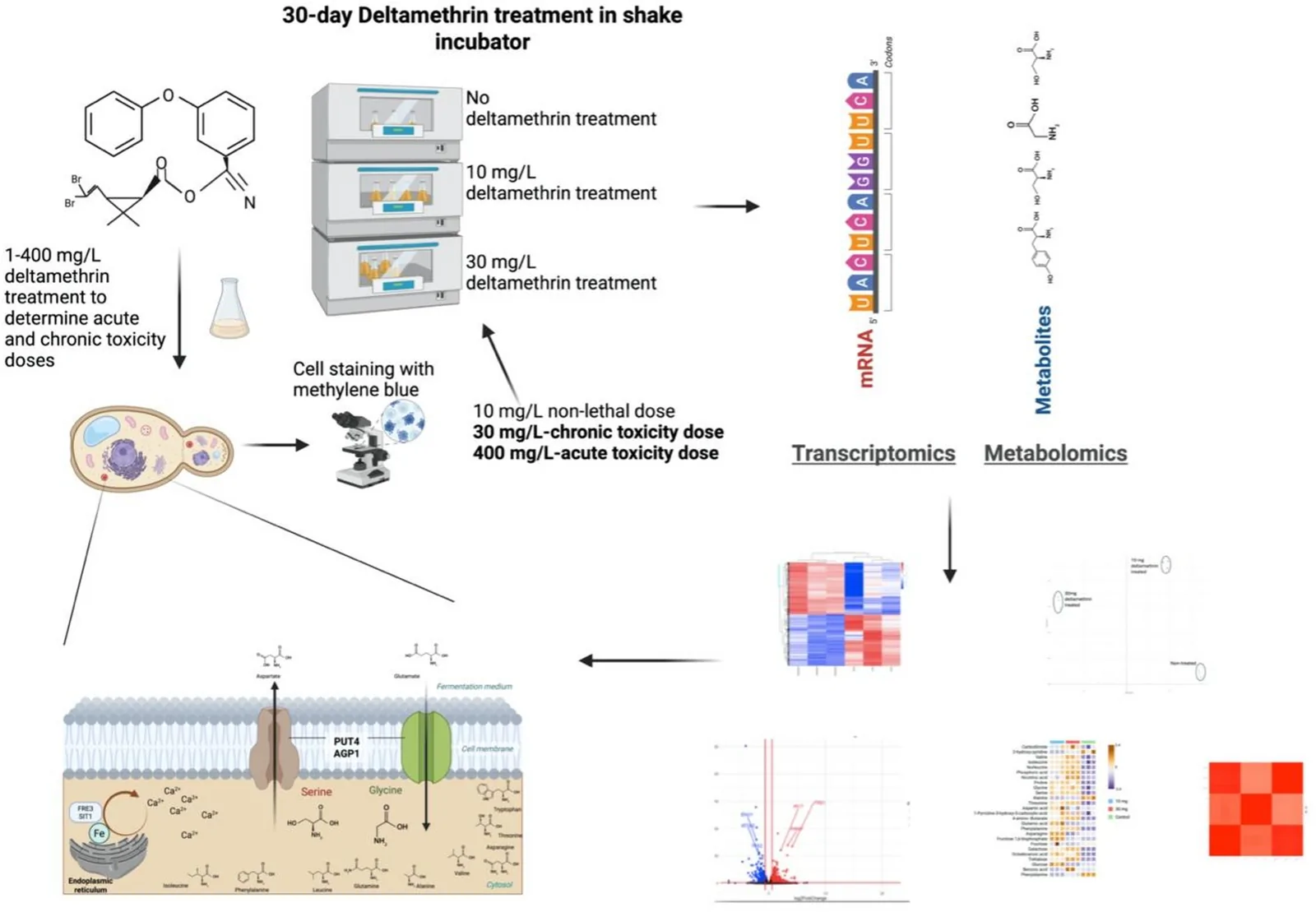
The experimental studies performed in this research to determine acute and chronic toxicity doses for *Saccharomyces cerevisiae*. DLM, ranging from 1 to 400 mg/L, was added to YPD medium when yeast cells were in the mid-exponential phase. After a 3-hour treatment with DLM, the cells were stained with methylene blue to distinguish between dead and alive cells under the microscope. Based on the count of dead and alive cells, the acute and chronic toxicity doses of DLM were determined. Following that, yeast cells were exposed to 10 mg/L and 30 mg/L DLM for 30 days. At the end of the 30-day DLM exposure, yeast cells were monitored through metabolomics and transcriptomics. The results were graphed to interpret the metabolic reprogramming in yeast in response to DLM exposure.

According to the European Food Safety Authority (EFSA), DLM was the fourth most frequently detected pesticide residue in food samples tested in 2020^12^. In 2022, EFSA’s coordinated surveillance based on 12 food groups across 30 European Union countries found that 34 food samples exceeded the Maximum Residue Limit (MRL) of DLM^13^. In line with that, EFSA recommended reconsidering MRL of DLM due to the lack of comprehensive toxicological evaluation in model organisms^14^. System toxicology provides insights into the ecotoxicological effects of DLM on model organisms to evaluate how DLM acts as a toxicant. Therefore, the perturbations in metabolism due to DLM exposure could enhance our understanding of prolonged repercussions of DLM. By means of wide range of omics tools, model organisms including zebrafish^15–19^, African clawed frog^20^, male mice^9^, crucian carp^21^, adipocytes, nematode^11,22^, mussel^23^, crab^24–26^, goldfish^27^, channel fish^28^, and rats^9,29,30^ were employed to unravel DLM toxicity. To the best of our knowledge, it is the first study that the chronic exposure of DLM was investigated in a non-pathogenic yeast *Saccharomyces cerevisiae* as a eukaryotic model organism at molecular level. Throughout this study, yeast cells were firstly screened microscopically using methylene blue staining to determine the non-lethal and chronic toxicity doses based on cell viability criteria (≥ 50% dead for acute, ≤ 10% dead for chronic). Yeast cells in flask were exposed to chronic toxicity dose and the non-lethal toxic dose for 30-day, separately. Following the exposure of both doses, untargeted metabolomics and transcriptomic profiling were conducted to elucidate the molecular mechanisms of DLM toxicity (Fig. 1). Prior to this study, model organisms exposed to DLM had shown ER stress and imbalanced iron hemostasis as cellular responses^31–35^. As a part of cellular response to DLM treatment, during ER stress, cells activate unfolded protein response mechanisms orchestrated by ER stress response genes, mainly inositol requiring enzyme (*IRE1*). The main goal of this mechanism is to fix unfolded proteins and mitigate the damage caused by impaired protein folding^33,35,36^. Here, in yeast metabolism, multi-omics approach combining metabolomics and transcriptomics presented evidence regarding the regulatory role of amino acids in response DLM-induced ER stress and imbalanced iron hemostasis.

## Materials and methods

### Cell culture and exposure experiments

*Saccharomyces cerevisiae* BY4742 (Matα; his3Δ1; leu2Δ0; lys2Δ0; ura3Δ0) from Euroscarf collection (Frankfurt, Germany) was used as a model organism. Preculture of the yeast was obtained through the inoculation from its 30% glycerol stock at −80 °C. *S. cerevisiae* was aerobically grown in YPD (Sigma-Aldrich, St. Louis, MO) medium (10 g/L yeast extract, 20 g/L peptone, and 20 g/L glucose) at 30 °C for overnight. To carry out cell viability in response to deltamethrin (Sigma-Aldrich, St. Louis, MO) exposure, 1% of the preculture was harvested through the centrifugation at 10000 rpm for 10 min and washed twice with sterile YPD medium before inoculation into 25 ml YPD medium in a 125 mL flask. Initial cell concentration was measured at 600 nm in Shimadzu UV-Vis 1800 spectrophotometer (Shimadzu Corporation, Kyoto, Japan). To find out the exact induction point for deltamethrin exposure, the fermentation, cellular growth was monitored to determine mid-exponential phase (OD = 0.8±0.05) (Supplementary Fig. 1) At the mid-exponential growth phase of cultivated *S. cerevisiae*, DLM stock solution (50 g/L) prepared in DMSO was added to culture medium at concentration ranging from 1 to 400 mg/L. The final concentration of DMSO in the assay was kept at 0.5% (v/v). The cultures containing 0.5% (v/v) DMSO without DLM were used as vehicle control. This concentration was chosen based on the previous studies demonstrating that DMSO does not induce any substantial physiological or transcriptomic alterations in yeast cells at this concentration or below^37–39^. Cellular growth was monitored for all treatment conditions, including the DMSO vehicle control (Supplementary Fig. 2). Cell viability after DLM treatment was evaluated via methylene blue staining, which was previously described^40^. Briefly, the aliquots of culture medium containing yeast cells exposed to 3-hour deltamethrin treatment was taken and mixed with the same volume of the solution containing 0.01% methylene blue and 2% sodium citrate. The microscopical examination of DLM-treated yeast and vehicle control cultures (DMSO without DLM) was carried out to observe blue-colored cells and white-colored cells as dead and alive, respectively. Dead and alive cells were counted, and the inhibition rate were calculated as previously described^41^. The images of dead and alive cells stained with methylene blue were illustrated in Supplementary Fig. 3.

### Metabolomics analysis

The metabolite extraction protocol was slightly different from previously described^42^. The extraction was performed with 0.5 mL treated w/o DLM yeast cells, which was harvested in the mid-exponential phase at its growth. The metabolomics analysis was performed by comparing DLM-treated samples with the DMSO vehicle control (DMSO without DLM) following 30 days of exposure. Before the metabolite extraction, harvested yeast cells in cryogenic vials were immediately submerged into nitrogen to keep cells for further of extraction, and stored at −80 °C. The cold methanol quenching was carried out by adding 2.5 ml methanol at −80 °C to 0.5 mL defrosted yeast cells. The mix was centrifugated at 4500 rpm for 5 min at 4 °C, and cell pellets were obtained by discarding supernatants. The cell pellets were dried under nitrogen flow using a nitrogen evaporator (Biotage Inc, North Carolina) under 25 °C for 2 h. Following that, 1 ml acetonitrile/water mixture (1:1, v/v) at 4 °C were added into dried cell pellets with glass beads. The mixture was vigorously mixed. After that, the centrifugation at 4500 rpm for 5 min at 4 °C was carried out to obtain 0.8 mL supernatant. The supernatant was dried under nitrogen flowing at 25 °C for 6 h. Before injecting samples for GC/MS analysis, the trimethylsilylation was performed to derivatize samples. 10 µL of 40 mg/mL methoxyamine chloride in pyridine (Sigma-Aldrich, St. Louis, MO) was added, and kept for 90 min at 200 rpm under 30 °C. The trimethylsilylation continued by adding 45 µL of N-methyl-N-trimethylsilyltrifluoroacetamide (Sigma-Aldrich, St. Louis, MO). The mixture was incubated at 30 °C with 200 rpm for 30 min. For GC/MS, an Agilent 7890 A GC/5975 C MSD system (Agilent Technologies, Santa Clara, CA) coupled with an HP-5MS capillary column (0.25 μm thickness, 0.25 mm diameter, 30 m length) was used. One microliter of the derivatized sample was injected into the GC inlet in split mode with a split ratio of 1:22. The oven temperature was first set to 50 °C for 1 min, then increased to 330 °C at a rate of 20 °C per minute, where it remained for 5 min. Mass spectra of samples were obtained within a scan range of 85–500 m/z using electron impact at 70 eV. The ion source and transfer line temperatures maintained at 230 °C and 280 °C, respectively. The raw data from the GC/MS analysis were analyzed using the AMDIS software for peak detection and mass spectral deconvolution^43^. After the processing, the data were uploaded to SpectConnect (http://spectconnect.mit.edu) for peak alignment and to generate a data matrix using the Golm Metabolome Database (GMD) mass spectral reference library^44,45^. Heatmap analysis was carried out in biorender.com. Principal component analysis (PCA) and PLS-DA model fitting were performed through R packages including mixOmics^46,47^. To find out the most affected pathways in yeast metabolism, the enrichment analysis for each dose was performed in MetaboAnalyst 6.0^48^.

### Transcriptomics analysis

Gene expression profiles of yeast cells exposed, and non-exposed DLM were obtained through the steps of RNA extraction, sequencing, and bioinformatic analysis of sequence data. Total RNA extraction protocol performed according to Qiagen RNeasy Mini Kit instructions. The cDNA library was constructed via Illumina TruSeq mRNA Sample Prep kit (Illumina, Inc., San Diego, CA, USA). Following that, sequencing performed in Illumina NovaSeq (Illumina, Inc., San Diego, CA, USA). Adaptor sequences and low quality reads were removed via Trimmomatic^49^. Reads were mapped to the *Saccharomyces cerevisiae* reference genome (NCBI Accession GCA_003086655.1) with Kallisto^50^. Differential gene expression analysis on the read counts were carried out by using DeSeq2^51^. Differential gene expression analysis was performed using the DMSO vehicle control as the reference group following 30 days of exposure. Graphical representations of volcano plots, enrichment analysis, and hierarchical cluster analysis were constructed through R packages^46^ while human homologs of DEGs were also enriched in MetaScape^52^.

### Reverse transcription quantitative PCR

*PUT4*, *FRT2*, *IRE1*, *AGP1*, *SIT1*, and *FRE3* were chosen for Reverse Transcription Quantitative PCR (RT-qPCR). Primers used in this study are listed in Supplementary Table 1. cDNA of yeast samples was obtained from total RNA extracted by means of QuantiTect Reverse Transcription Kit (Qiagen, Valencia, CA), following the manufacturer’s protocol. RT-qPCR was conducted with the LightCycler FastStart DNA Master PLUS SYBR Green I Kit (Roche Molecular Biochemicals, Mannheim, Germany), according to the manufacturer’s guidelines. All samples were analyzed in triplicate, and fold changes were calculated using the 2^−ΔΔCt^ method based on the average results, as described previously^53^. Statistical differences were found by running one-way ANOVA followed by Tukey’s multiple-comparison test. *P* values of six genes based on the statistical analysis were collated in Supplementary Table 2.

## Results and discussion

### Determination of acute and chronic DLM doses

Baker`s yeast (*Saccharomyces cerevisiae*) has been a model organism for ecotoxicological assays of certain chemicals due to easy-to-grow, non-pathogenic, and being innate resident of foods^54,55^. Here, the toxicity assays of DLM to find out chronic and acute toxic doses were set 1, 10, 20, 30, and 40 mg/L based on a conducted research on the DLM exposure of *Saccharomyces cerevisiae*^56^. The DLM treatment was carried out when yeast cells in the fermentation medium were at the mid-exponential growth phase, which was determined by the yeast cellular growth that was shown in Supplementary Fig. 2A. Microscopic imaging of methylene blue-stained yeast cells treated with 1 and 10 mg/L DLM showed no indication of cell death after the treatment. Based on the microscopic images of methylene-blue dyed yeast cells after DLM treatment, the toxic effect of DLM treatment became evident at 20 mg/L DLM, which was consistent with previous findings^56^. There were, however, slight differences in the ratio of dead to viable cells after DLM treatment. The differences could arise from the toxicological assessment methods used in that study. While we employed methylene blue staining following a 3-hour incubation with DLM exposure^40^, the plate counting after 2 h of DLM exposure was performed in the previous study^56^. A comparative analysis of *S. cerevisiae* toxicity assays between plate counting and methylene blue staining for H₂O₂, menadione, and allyl alcohol treatments showed that plate counting detected higher percentage of dead cells than did methylene blue staining^57^.

**Fig. 2:**
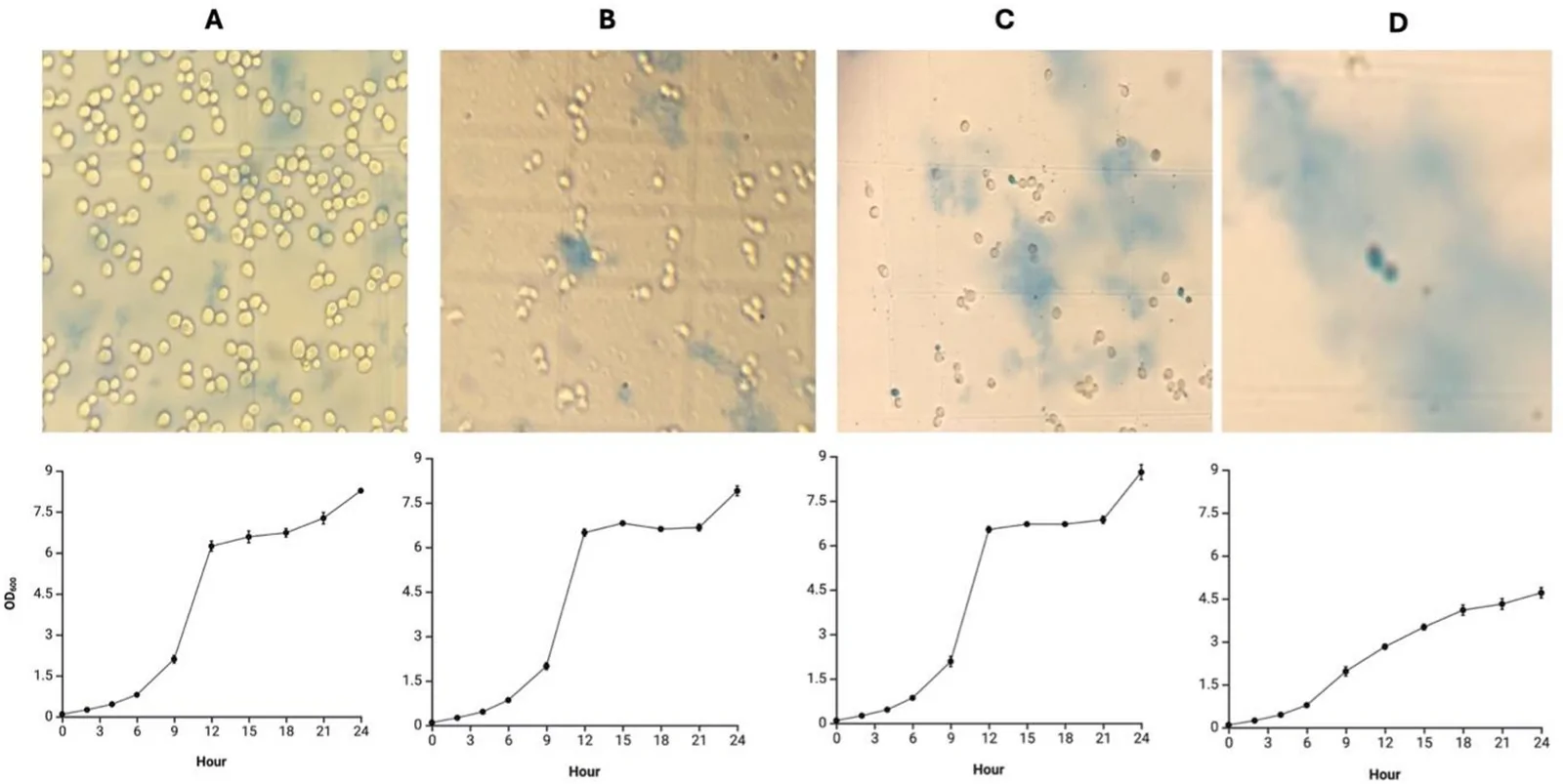
The microscopic images of stained *S. cerevisiae* BY4742 cells and the 24-hour growth curves of *S. cerevisiae* BY4742 are shown for different treatments: without DLM treatment (**A**), with 10 mg/L DLM treatment (**B**), with 30 mg/L DLM treatment (**C**), and with 400 mg/L DLM treatment (**D**).

The higher ratio of dead cells at 30 mg/L and 40 mg/L DLM treatment as compared to the 20 mg/L DLM treatment were found in the microscopic evaluation. Based on the tested concentrations ranging from 1 to 40 mg/L DLM, 30 mg/L DLM reached the chronic toxicity threshold (≤ 10% dead cells), while 10 mg/L DLM was the non-lethal dose based on methylene blue staining of DLM-exposed yeast cells (Supplementary Table 3). From there on, 30 mg/L and 10 mg/L DLM were set as the chronic and non-lethal toxicity doses, respectively. No differences in cellular growth were observed between DMSO vehicle control and untreated cultures (Figure S2), indicating that the DMSO concentration used in this study did not show any significant effect on growth of yeast cells under the experimental conditions. Since the acute toxicity in yeast was previously reported at 100 mg/L DLM^56^, additional tests were conducted at 100, 120, and 140 mg/L. However, these concentrations did not reach the acute toxicity threshold (≥ 50% dead cells) in our hands. To further examine the acute toxicity, yeast cultures were exposed to 150–400 mg/L DLM, and cellular growth was monitored at each concentration (Supplementary Fig. 2B). Acute toxicity was microscopically confirmed at 400 mg/L DLM treatment. Microscopic images of dead and alive cells, and the growth curves of yeast exposed to chronic toxicity, acute toxicity, and non-lethal doses were shown in Fig. 2.

### Metabolome profiles of DLM-treated yeast cells

**Fig. 3:**
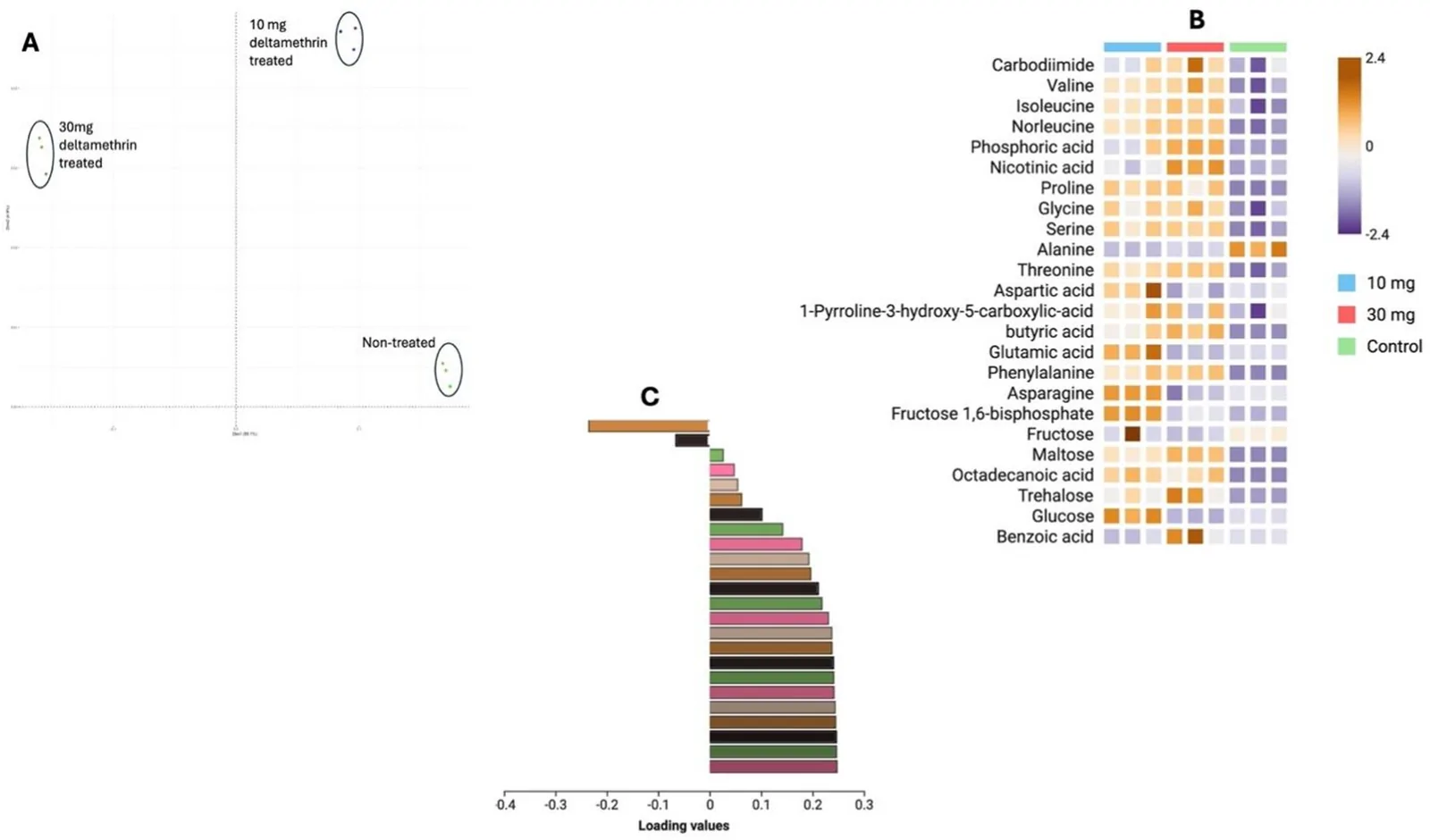
A Principal Component Analysis (**A**) of intracellular metabolites of *S. cerevisiae* BY4742 exposed to 10 mg/L and 30 mg/L DLM, non-exposed to DLM, a heat map (**B**) of identified metabolites, loading values (**C**) of metabolome of yeast strains based on PLS-DA.

Toxicological assessments of DLM via metabolomics have been conducted in several model organisms, including zebrafish^1^, *Xenopus laevis*^20^, male mice^10^, and crucian carp^58^. To investigate the metabolic changes in *Saccharomyces cerevisiae* exposed to DLM, we performed untargeted metabolomics analysis on yeast treated with 10 mg/L and 30 mg/L DLM, alongside DMSO vehicle-treated yeast as the control group. The raw spectral data yielded 285 peaks, among which 25 metabolites were identified using a Golm Human Metabolome database^45^. Amino acids represented the most abundant metabolite group found in the metabolomic analysis, consistent with previous reports on the effects of pyrethroids` exposure on amino acid metabolism^59,60^. Principal Component Analysis (PCA) score plots clearly separated the metabolomes of DLM-treated and DLM-untreated yeast cells. The most distinguished metabolome was found as the metabolome of yeast-treated with 10 mg/L DLM (Fig. 3A). A similar pattern was noted in *X. laevis*, whose exposure to sublethal DLM led to noticeable metabolomic shifts in its metabolome^20^, suggesting that even non-lethal doses of DLM with non-detected phenotypic changes due to the DLM exposure could significantly affect organisms` metabolome.

Heatmap analysis revealed that several amino acids—including isoleucine, norleucine, proline, glycine, serine, and threonine—accumulated in DLM-treated yeast regardless of the concentrations applied in this study. Conversely, phenylalanine and alanine levels were reduced in DLM-treated yeasts as compared to non-treated yeast. The intracellular valine was elevated only in the 30 mg/L DLM treatment group, while aspartic acid, glutamic acid, and asparagine levels were markedly increased in the 10 mg/L DLM treatment group. In terms of carbohydrate metabolism, trehalose accumulation was observed in yeast exposed to 30 mg/L DLM, whereas fructose 1,6-bisphosphate levels rose in cells treated with 10 mg/L DLM (Fig. 3B). Intracellular trehalose accumulation was previously reported as metabolic response in yeast to enhance resilience while tolerating stressors^61^. Trehalose accumulation was reported in *Tetrahymena thermophila* (an aquatic protozoon) following exposure to the type II pyrethroid β-cypermethrin^60^, indicating that different model organisms could react in a same way to type II pyrethroids exposure. Partial Least Squares Discriminant Analysis (PLS-DA) further highlighted the metabolic reprogramming in the face of DLM exposure. The constructed model of this study yielded R²X = 0.885, R²Y = 0.981, and Q² = 0.961, which point out high reliability and predictable strength of the model (Supplementary Fig. 4). The loading values of the metabolites obtained from the model facilitate to interpret the effect of metabolites on the separation between treated and non-treated groups^62^. The loading values of most metabolites were positively correlated with PC1, while only fructose and alanine showed negative correlations (Fig. 3C). Variable importance in projection (VIP) scores provide a list in descending order of the metabolites for enhancing PLS-DA model`s discriminating ability between groups. Metabolites having VIP > 1 are considered to contribute to the model significantly^30^. VIP scores of 12 metabolites including threonine, norleucine, phenylalanine, serine, isoleucine, alanine, proline, valine, octadecanoic acid, glycine, phosphoric acid, and trehalose were higher than 1 based on the PLS-DA model, which suggested that those 12 metabolites in yeast metabolism could be biomarker metabolites in response to DLM treatment regardless of the applied concentrations (Supplementary Table 4). Previously, metabolites meeting the criteria of VIP > 1, fold change differences (*p* < 0.05), and fold change > 1 of heatmap analysis were considered as key biomarker metabolites^63^. Based on these criteria, it was proposed that isoleucine, norleucine, proline, glycine, serine, and threonine as biomarker metabolites in this study. The amino acid metabolism of yeast exposed to 10 mg/L DLM was significantly affected, while serine and alanine pathways prominently altered regardless of the applied dose. Interestingly, alanine was the most negatively correlated metabolite with PC1 in the PLS-DA model (Fig. 3C). Similar disruptions in serine and alanine metabolism were reported in mice treated with type I pyrethroid bifenthrin and type II pyrethroid lambda-cyhalothrin^64^, as well as in earthworms exposed to cypermethrin^59^.

It was pointed out that the regulation of amino acids within the cell could be related to ER stress that can be triggered by generated reactive oxygen species as a response to oxidative stress caused by xenobiotic exposure^65^. Rats and hy926 human endothelial cell having homocysteine-induced ER stress administered glycine and serine to alleviate the stress^66^. Similarly, weaning piglets had damage in intestinal barrier that disturbs nutrient assimilation due to the weaning-induced ER stress. Glycine and serine administration before weaning reduced the damage in intestinal barrier, suggesting that two amino acids had a regulatory role to mitigate ER stress^67,68^. In this study, the intracellular levels of serine and glycine of 10 mg/L and 30 mg/L DLM-exposed yeast were higher than that of control, implying that serine and glycine could act together to relieve ER stress caused by DLM exposure. Also, it was reported that not only glycine and serine, but also cysteine, glutamic acid, and glycine via the production of glutathione, as a molecule to be utilized against oxidative stress, mitigated ER stress in epithelial cells^65^. The metabolomics analysis showed that intracellular glutamic acid was only high in yeast treated with 10 mg/L DLM even though 30 mg/L DLM was considered as a toxic dose for yeast.

**Fig. 4:**
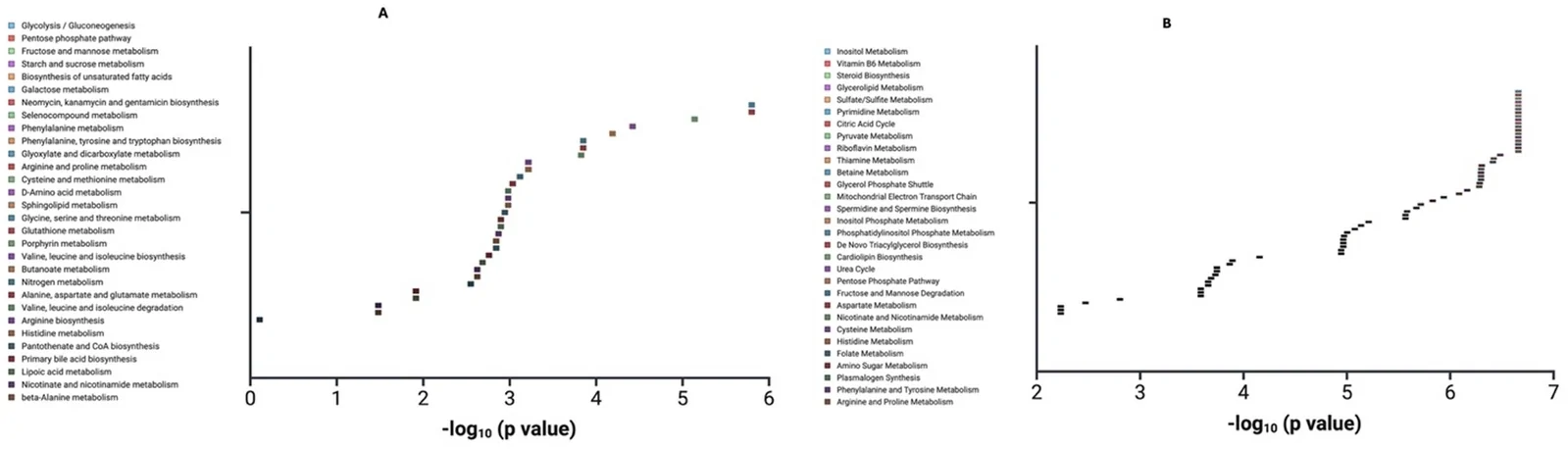
Based on the metabolomics analysis of DLM-(not) treated yeast cells, the enrichment analysis of 10 mg/L (**A**) and 30 mg/L (**B**) DLM-treated yeast cells is shown.

Pathway enrichment analysis based on the metabolomics data provided insight into the biological processes affected by DLM exposure. At 10 mg/L DLM exposure, carbohydrate and amino acid biosynthetic pathways were mainly affected (Fig. 4A), while 30 mg/L DLM exposure predominantly influenced lipid metabolism (Fig. 4B). A metabolomics analysis that was conducted on crucian carp brain exposed to DLM found that amino acid biosynthesis was one of the most affected metabolisms^21^, which is consistent with the findings in this study. On the other hand, glycerolipid metabolism was the most affected pathway in yeast treated with 30 mg/L DLM. That aligns with previous studies reporting alterations in glycerophospholipid levels in zebrafish, human and *Anopheles sinensis* following DLM exposure^1,69–71^. Glycerol-3-phosphate, a key metabolite in glycerophospholipid pathway, was found to accumulate in DLM-treated *Anopheles sinensis*^71^, corroborating the finding of this study in which glycerophosphate metabolism was highly disrupted at 30 mg/L DLM exposure. Also, it was found that the change in lipid metabolisms and energy metabolism were associated with neurodevelopmental diseases^69^.

### Transcriptome of yeast exposed to DLM at lethal and non-lethal doses

**Fig. 5:**
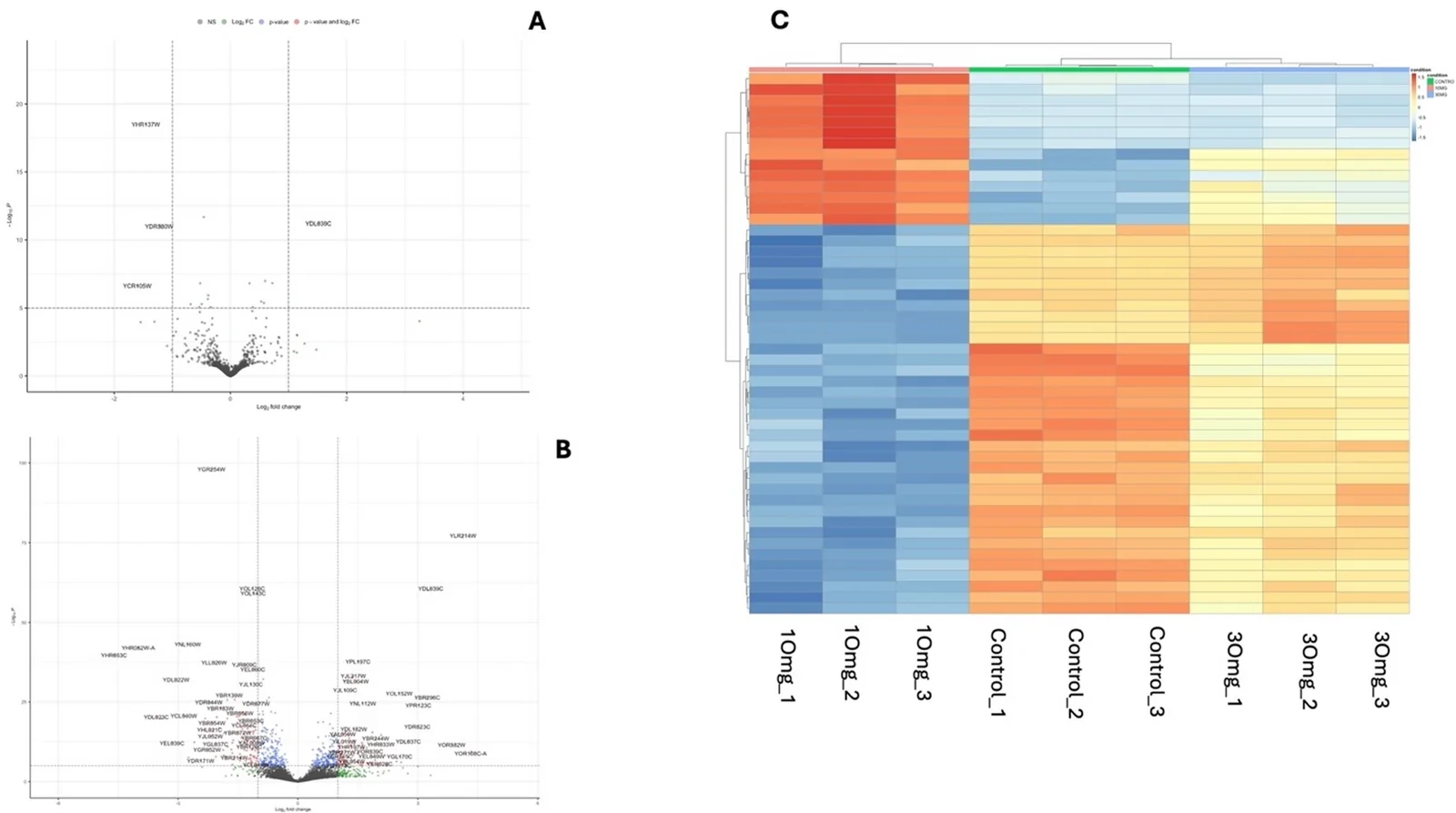
The volcano plots indicate the distributions of differential genes based on the comparisons of the treatment. The comparison between 30 mg DLM treatment and non-treatment (**A**), and the comparison between 10 mg DLM treatment and non-treatment (**B**) are illustrated. Hierarchically clustered heatmap of top 50 genes differentially expressed is presented to visually demonstrate (**C**).

**Fig. 6:**
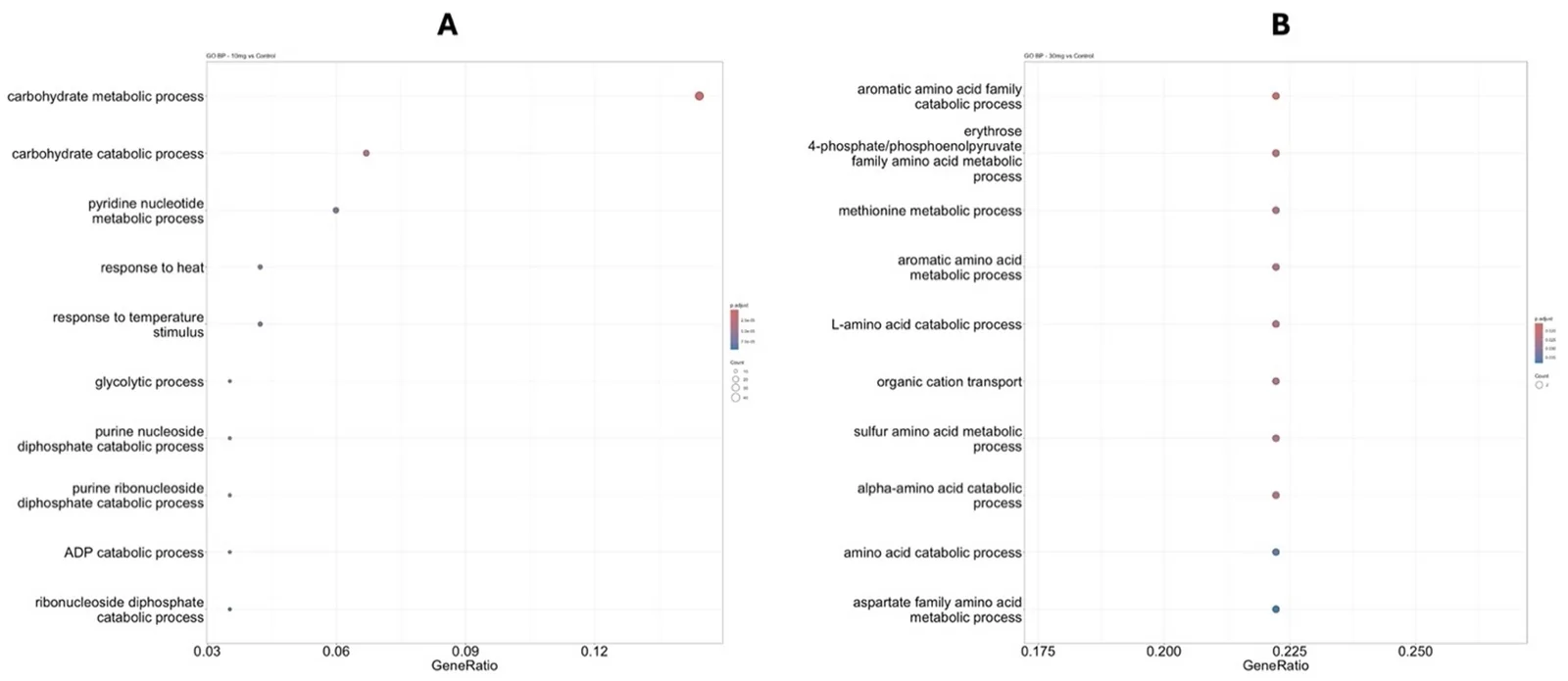
The enrichment analysis of 10 mg/L (**A**) and 30 mg/L (**B**) DLM treated yeast cells is shown.

High-throughput transcriptomic analysis of an organisms in response to a stressor enables to identify the alterations in gene expression levels^24,26,54,55^. This approach has been extensively used to investigate the molecular mechanisms underlying DLM toxicity in various species, including zebrafish^15,17,18^, adipocytes, nematode^11,22^, crucian carp^21^, mussel^23^, crab^24–26^, goldfish^27^, channel fish^28^, and rats^9,29,30^. In this study, differential expression analysis of each treatment group as compared to control group was performed in Deseq2, resulting in differentially expressed genes illustrated as volcano plots in Fig. 5A and B. The magnitude of change in the transcriptome of 30 mg/L DLM-treated yeast was lower than that of 10 mg/L DLM-treated yeast when compared to the DMSO vehicle control group. The number of differentially expressed genes in yeast treated with 10 mg/L DLM were found 110, however, 15 genes` expressions in the transcriptome of yeast were regulated in the presence of 30 mg/L (|Log_2_ FC > 1| and *p* < 0.05). It was interesting to note that 97 genes were upregulated in yeast treated with 10 mg/L DLM while only four genes including *DUR3*, *PRM7*, *BSC1*, and *AGP1* expressions of yeast treated with 30 mg/L DLM were upregulated. Gene Ontology (GO) analysis of the differentially expressed genes provided insights into their molecular functions, as detailed in the Supporting Information (SI1). DLM exposure in 3T3-L1 adipocytes led to the upregulation of *AMPKα*, a carbohydrate metabolism regulator^35^. In line with that, the enrichment analysis of 10 mg/L DLM indicated that carbohydrate metabolisms were highly regulated (Fig. 6A), which was corroborated by the metabolomics analysis of this study. The increased expression of *MAL32*, *MAL12*, and *IMA5* of carbohydrate metabolism at 10 mg/showed the enhanced carbohydrate hydrolysis. Also, transcriptomic analysis of 30 mg/L DLM-treated yeast pointed out that the most affected pathways involved in amino acid metabolism (Fig. 6B), suggesting that energy metabolism in yeast was influenced by DLM treatment.

**Fig. 7:**
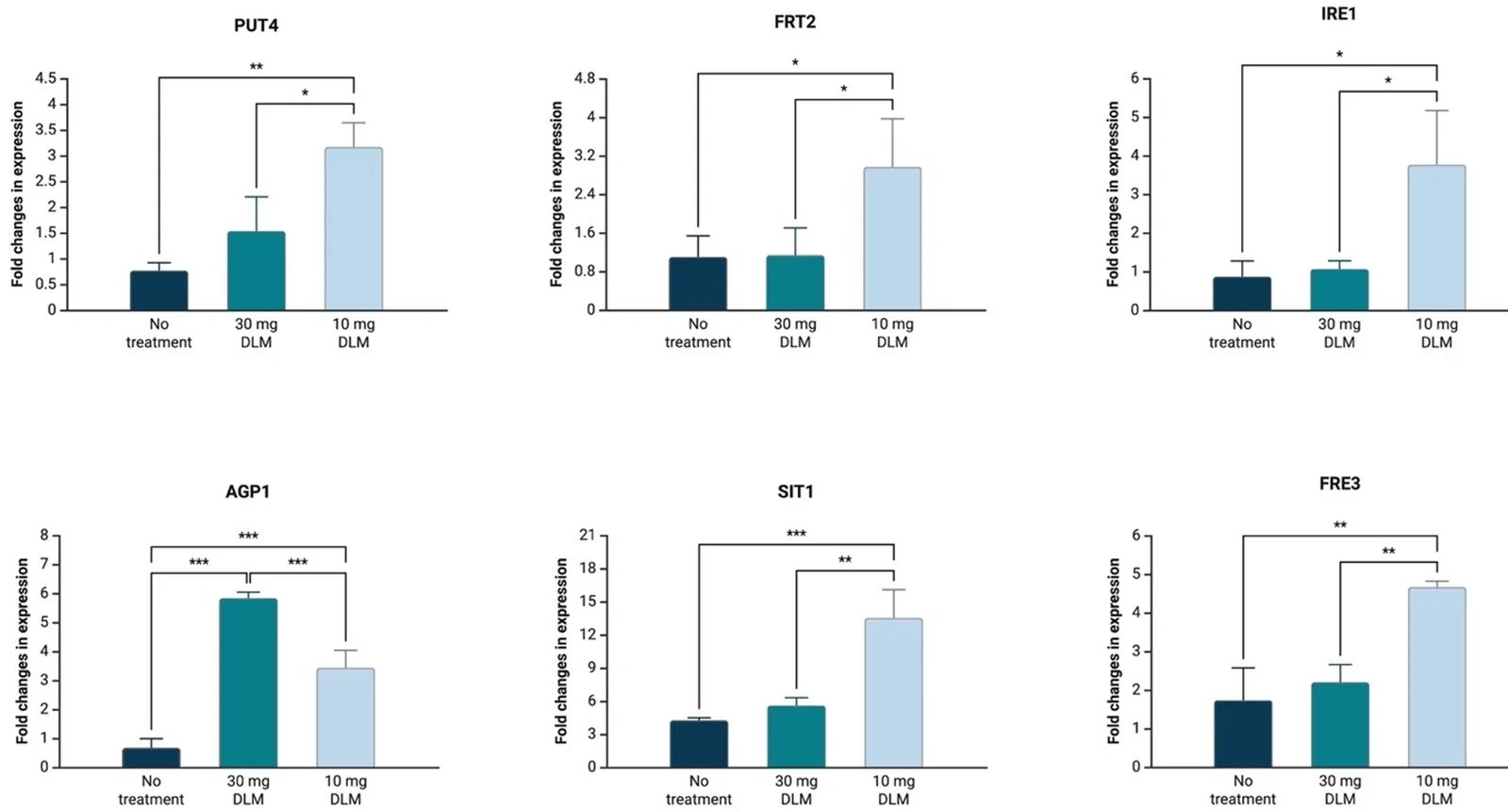
RT-qPCR data of six genes including *PUT4*, *FRT2*, *IRE1*, *AGP1*, *SIT1*, and *FRE3* are presented as mean value and standard deviations of three independent biological replicates. Statistical differences are indicated in asterisk that represent *P* value ranges (*P < 0.05, **P < 0.01, ***P < 0.001).

In this study, ER-located genes including *CSM4*, *MUM3*, *BIO5*,* ERG25*,* TOH1*,* PAU12*, and *FRT2* were differentially expressed in the presence of 10 mg/L DLM. *CSM4*, *MUM3*, *PAU12*, and *BIO5* of yeast-treated with 10 mg/L DLM were upregulated. It has been known that ER-located transmembrane proteins can also sense ER stress^72^. ER facilitates protein folding so that cellular functions can be properly maintained. Several studies have shown that DLM exposure induces ER stress by promoting Ca^2+^ release into the cytosol and activating the unfolded protein response mechanism^11,21,33,35,36^. As a part of the unfolded protein response mechanism, *IRE1* gene is upregulated to assist chaperons to efficiently perform protein folding. *IRE1* protein mediates *HAC1* mRNA splicing by removing the intron of *HAC1* mRNA. Therefore, the unfolded protein response can be initiated after ER stress induced by stressors^73,74^. RT-qPCR results showed that 10 mg/L DLM treatment increased the expression of *IRE1* while no differences were found between no treatment and 30 mg/L DLM treatment (Fig. 7), suggesting that 10 mg/L DLM treatment led to ER stress in yeast. However, the chronic exposure of 30 mg/L DLM showed no indication of ER stress, which might occur due to the adaptation to DLM exposure. *PRM7* gene that was previously associated with increased lifespan in wild-type yeast strains^75,76^ was upregulated in transcriptomics analysis of yeast exposed to 30 mg/L DLM. The upregulation of *PRM7* pointed out possible adaptive responses to chronic DLM exposure at a toxic dose. Previous studies in other models showed that DLM toxicity induced the generation of reactive oxygen species, which triggered Ca^2+^ release from the endoplasmic reticulum (ER) into the cytosol^35,65,69,74^. As a part of ER stress, DLM-exposed cells needed to adapt acidic condition created by Ca^2+^ influx to the cytosol. *FRT2* upregulation that is associated with Ca^2+^ stress response and adaptation to acidic conditions in yeast^77^ was upregulated in yeast treated with 10 mg/L DLM and confirmed by RT-qPCR (Fig. 7).

DLM exposure in organisms resulted in also iron deprivation response that could induce the increase intracellular Ca^2+^ through signaling pathways^78–80^. Iron is an essential micronutrient for growth in yeast to maintain cellular activities including being a cofactor for enzymes and having indirect participations into oxygen related metabolisms^64,81,82^. In the face of iron depletion, depleted irons are compensated via extracellular siderophores that chelate iron to form siderophore-iron complexes. Towards that end, yeast utilizes two distinctive metabolic routes to transport siderophore-iron complexes into cells to supply iron into cells. Transcriptomic analysis of yeast treated with 10 mg/L DLM demonstrated that bot metabolic routes were activated through *FRE3* and *SIT1* upregulations. In the first metabolic route of siderophore-iron complexes` uptake during iron deprivation, the bioavailability of iron is provided by the reduction of siderophore-iron complexes. The reduction occurs in the cell membrane equipped with metalloreductases that catalyze the transformation of siderophore-iron complexes to ferrous state of iron. Subsequently, the uptake of ferrous ion into cells occurs via membrane transporters^82^. The reduction process of siderophore-iron complexes is performed by metalloreductase genes of members of *FRE* family in yeast genome. Plasma membrane localized *FRE3*, as a member of metalloreductase genes, carries out the reduction of hydroxamate-iron siderophores to generate ferrous state of iron for its uptake into cell^81,83^. The upregulation of *FRE3* gene in response to iron deprivation was reported^64,83^. Here, *FRE3* gene was upregulated in yeast treated with 10 mg/L DLM, and confirmed by RT-qPCR (Fig. 7).

In the second metabolic route for ferric siderophores` uptake, plasma membrane-bounded *SIT* family transporters are employed for the intact transportation of ferric siderophores into cytosol. Following the transportation of ferric siderophores into cell, iron ions are released by cleaving the bond between iron and siderophores^64,83^. Each of *SIT* family transporters show specific ferric siderophores preference to carry into cell^83^. Ferrioxamine B^84^ and ferrichromes are known specific ferric siderophores for *SIT1* transporter^85^. In the absence of specific ferric siderophores, *SIT1* protein are directed towards vacuole for its degradation in lieu of its localization into yeast plasma membrane for ferric siderophores` uptake. Thus, ferric siderophores` specific transportation feature of *SIT1* protein facilitates to prevent the permeability of potential toxic compounds. Previous studies pointed out the upregulation of *SIT1* in response to iron deprivation^64,83,85^. In this study, plasma membrane localized *SIT1* were upregulated in yeast treated with 10 mg/L DLM, which was confirmed by RT-qPCR (Fig. 7). In the light of the results of the transcriptomics analysis and RT-qPCR, DLM treatment at 10 mg/L DLM led to iron deficiency response, and the response availed of iron uptake mechanisms in yeast. A study conducted on ER stress-induced yeast strain showed that iron did not trigger and/or cause ER stress. On the contrary that, iron bioavailability mitigated ER stress^73^.

### Intertwining of metabolomic and transcriptomic response to DLM exposure

The decline in iron levels of rats exposed to 17.4 mg DLM/kg body weight for 9 weeks was previously reported due to the disruption in iron hemostasis of cell^79^. In the metabolomics analysis, branched chain amino acids such as valine and isoleucine were found higher as compared to the control group regardless of the applied DLM concentrations in this study. The decreases in those amino acids were previously correlated with biomarkers of iron deficiencies^32^. The increases in those amino acids in yeast treated with DLM was found in metabolomics analysis. Their levels in yeast treated with 10 mg/L DLM were lower than those in yeast treated with 30 mg/L DLM. Transcriptomics analysis pointed out the upregulations of iron deprivation response genes in yeast treated with 10 mg/L DLM whereas the transcriptome of yeast treated with 30 mg/L DLM did not show any signs of iron deprivation response due to the DLM treatment. One possible explanation for the significantly reduced number of differentially expressed genes in response to prolonged exposure to 30 mg/L DLM is the development of an adaptive stress response. Rather than showing reduced biological activity, chronic exposure to the higher DLM concentration may have stimulated transcriptional reprogramming toward a new homeostatic state, resulting in reduced number of genes remaining differentially expressed at the end of the 30-day exposure period. These adaptive responses have been discussed in the context of hormesis, in which exposure to low or sustained stress levels induces cellular defense mechanisms that enhance stress tolerance and restore cellular homeostasis instead of maintaining prolonged transcriptional activation^86,87^. Similarly, genome-wide transcriptional changes have been observed in *Saccharomyces cerevisiae*, where cells initially activate a large environmental stress response that is subsequently decreased as adaptation mechanism^88,89^. Taken together, we suggest that this interpretation should be considered as a possible biological explanation rather than direct evidence of hormesis since the present study involves only a single endpoint in response to chronic exposure to DLM.

The intracellular interactions between Ca^2+^metabolism, ER stress, and iron toxicity in yeast were reported in previous studies^32,34,73^. At high intracellular concentrations of iron, reactive oxygen species are produced through Fenton reactions, leading to interruptions of cellular processes and damages in cellular components. To ameliorate iron toxicity, Ca^2+^influx is employed to survive high iron-induced ferroptosis-cell death due to in vivo iron accumulation^32,34,73,90^; The role of Ca^2+^ to mitigate the iron-induced toxicity in yeast was elucidated in yeast strain with *MID1* and *CCH1* genes deletions. *MID1* and *CCH1* genes provide Ca^2+^ permeability in plasma membrane of cell^34^. The yeast strain exposed to 10 mM Fe^2+^ was rescued from iron toxicity by the supplementation of 10 mM Ca^2+^, suggesting that Ca^2+^ influx alleviated iron toxicity^34^. Elevated levels of glutamic acid and aspartic acid of yeast treated with 10 mg/L DLM implied a potential cellular response to Ca^2+^-induced charge imbalance. Also, transcriptomic responses to 10 mg/L and 30 mg/L DLM exposure led to *PUT4* and *AGP1* amino acid permeases` upregulations, respectively. *AGP1* gene in yeast partakes in amino acid transportation into cell, especially for uncharged amino acids including serine, threonine, asparagine, glutamine, and cysteine^91^. The accumulations of isoleucine, norleucine, proline, glycine, serine, and threonine, and the decrease in aspartic acid and asparagine was found in the metabolomics analysis of yeast treated with 30 mg/L DLM, suggesting that amino acid transportation happened due to the work of *AGP1* upregulation. Also, another amino acid permease *PUT4* that is responsible for glycine and proline transportation into the cell^92^ was upregulated in yeast treated with 10 mg/L DLM. Alleviating effects of glycine and serine in ER stress was previously shown^65–68^. In our hands, we did not find any regulations of Ca^2+^ influx genes. Therefore, we hypothesized that iron deprivation response could be managed by amino acid trafficking found in the metabolomics analysis.

Also, it was claimed that iron could have initiative effects on unfolded protein response to ER stress^73^. ER stress-induced yeast cells were screened for clustering of Ire1 proteins that requires oligomerization and phosphorylation to initiate the unfolded protein response to ER stress^32,34,73^. Poor Ire1p clustering was observed in yeast strains lacking *FRE* genes that is related with iron homeostasis. Ire1p clustering was improved at the medium containing high iron, suggesting that the activation of unfolded protein response could be achieved by iron uptake^73^. The further screening by transcription activators tagged with green fluorescent protein indicated the alterations in the expressions of heme-dependent genes in ergosterol biosynthesis. Also, the sterol content in ER stress cell was reported lower as compared to non- stressed cells^73^. It was speculated that sterol composition in cell membrane played a crucial role to regulate *IRE1* clustering, suggesting that iron uptake indirectly relieved cells from ER stress. In the transcriptomics analysis of yeast treated with 10 mg/L DLM, *ERG25* gene was found as downregulated. The enrichment analysis of yeast treated with 10 mg/L DLM corroborated the changes in lipid metabolism. Taken together, metabolomics and transcriptomics analysis pinpointed the crosstalk and reciprocity between ER stress and iron deprivation response in yeast.

## Conclusion

**Fig. 8:**
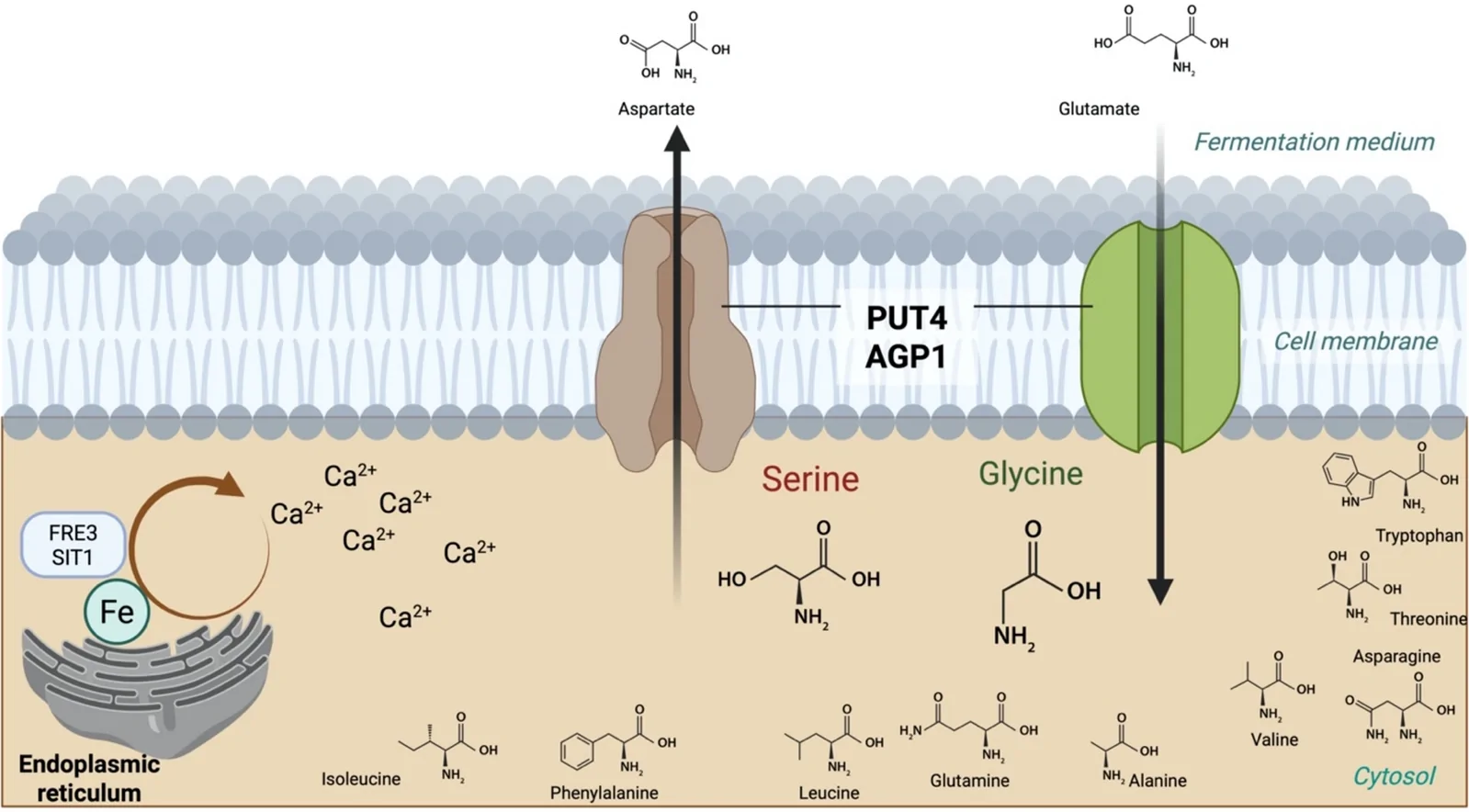
The illustration of intracellular process of cellular response to 10 mg/L DLM treatment.

The mechanisms of DLM-induced toxicity have been reported in various model organisms, yet deltamethrin mode of action at molecular level in yeast remains unclear. In this study, *Saccharomyces cerevisiae* was used to gain further insight into metabolites and gene expressions that were involved in the response to chronic DLM exposure for 30 days. Both omics approaches confirmed that yeast metabolism were more influenced when 10 mg/L DLM was applied as compared to 30 mg/L DLM. In this study, exposure to 10 mg/L DLM triggered Ca2 + influx from the ER, disrupting intracellular charge balance. In response to that, yeast modulated amino acid transport to help restore electrochemical homeostasis within the cell. Additionally, components of the cell wall were dynamically remodeled through altered metabolic pathways, indicating rewiring of the plasma membrane under DLM stress (Fig. 8). Disease enrichment analysis was performed using MetaScape based on the human homologs of differentially expressed yeast proteins identified following deltamethrin exposure. Although the experimental model in this study was *Saccharomyces cerevisiae*, the enrichment results indicate that the conserved molecular pathways perturbed by deltamethrin are significantly associated with a range of human diseases and clinical conditions. Notably, the enriched disease categories include neurological disorders (e.g., encephalopathies, tremor, dementia, peripheral nervous system diseases), cardiovascular conditions (e.g., myocardial ischemia, cardiomyopathies, cerebral infarction), metabolic dysfunctions (e.g., experimental diabetes mellitus, fatigue, muscle weakness), and mitochondrial and neurodegenerative diseases (Supplementary Fig. 5). These associations suggest that chronic or repeated deltamethrin exposure may have the potential to contribute to molecular alterations linked to these disease processes, through conserved stress-response and metabolic adaptation mechanisms. The results do not imply direct causality but rather highlight disease-relevant pathways that may be affected by deltamethrin exposure, underscoring the relevance of yeast as a translational model for assessing long-term human health risks.

This study demonstrated that yeast could serve as a valuable eukaryotic model for investigating the mechanistic underpinnings of DLM toxicity, offering insights into its broader biological impact at the molecular level. These findings highlight the value of metabolomic and transcriptomic profiling in understanding DLM toxicity and support its use in extrapolating health risk assessment.

## Supplementary Information

Below is the link to the electronic supplementary material.


Supplementary Material 1


## Data Availability

The RNAseq dataset generated and/or analysed during the current study is uploaded to a NCBI Bioproject (Accession number: PRJNA1393177) and accessible through “https://www.ncbi.nlm.nih.gov/sra/PRJNA1393177”.
