# Supplementary material for: A multi-omics systems toxicology framework reveals amino acid–mediated stress adaptation to deltamethrin exposure in a yeast model
Source: Sci Rep. 2026 Sep 1;16:27411. doi: 10.1038/s41598-026-66841-2 (PMC13534689; doi:10.1038/s41598-026-66841-2)
Supplement: Supplementary file 1 [file 41598_2026_66841_MOESM1_ESM.docx]

**A Multi-Omics Systems Toxicology Framework Reveals Amino Acid–Mediated Stress Adaptation to Deltamethrin Exposure in a Yeast Model**

Mustafa Yavuz^1,2^, Hakime Gul Yavuz^1^, Aytac Dursun Oksuzoglu^2^, Ceyhun Bereketoglu^2^, Adil Mardinoglu*^3,4^, Beste Turanli*^2,5^

^1^Denizli Food Control Laboratory Directorate, Ministry of Agriculture and Forestry, Denizli, Türkiye

^2^Department of Bioengineering, Marmara University, Istanbul, Türkiye

^3^ HealthCentre for Host-Microbiome Interactions, Faculty of Dentistry, Oral & Craniofacial Sciences, King’s College London, London SE1 9RT, UK

^4^Science for Life Laboratory, KTH—Royal Institute of Technology, SE-17165 Stockholm, Sweden

^5^Biotechnology Common Application and Research Center of Excellence (SABIOTEK), Istanbul, Türkiye

*Equal Correspondence:

Beste Turanli

*E-mail:* beste.turanli@marmara.edu.tr

Adil Mardinoglu

*E-mail:* adilm@scilifelab.se

**Supplemantary Figure 1**: The fermentation profile for cell growth to determine mid-exponential phase (OD=0.8±0.05), indicating the exact induction point for deltamethrin exposure.

**A**

OD

| Hours | Control | 1 mg/L deltamethrin | 10 mg/lL deltamethrin | 20 mg/L deltamethrin | 30 mg/L deltamethrin | 40 mg/L deltamethrin |
| --- | --- | --- | --- | --- | --- | --- |
| 0 | 0.2295 | 0.2575 | 0.234667 | 0.2575 | 0.2245 | 0.229 |
| 2 | 0.323 | 0.333 | 0.328 | 0.333 | 0.323 | 0.323 |
| 4 | 0.675 | 0.709 | 0.703333 | 0.709 | 0.685 | 0.665 |
| 6 | 0.717 | 0.819 | 0.749 | 0.819 | 0.814 | 0.717 |
| 9 | 2.406 | 2.115 | 2.34333 | 2.115 | 2.3 | 1.8 |
| 12 | 4.648 | 3.804 | 4.21933 | 3.804 | 3.427 | 2.971 |
| 15 | 5.45 | 5.05 | 5.2 | 5.05 | 4.7 | 4.25 |
| 18 | 6.15 | 5.9 | 6.06667 | 5.9 | 5.67 | 5.45 |
| 21 | 7.45 | 6.7 | 7.30667 | 6.7 | 6.76 | 6.765 |
| 24 | 8.095 | 7.87 | 8.09 | 7.87 | 8.1 | 7.58 |

**B**

OD

| Hours | Control | 150 mg/lL deltamethrin | 200 mg/L deltamethrin | 250 mg/L deltamethrin | 300 mg/L deltamethrin | 400 mg/L deltamethrin |
| --- | --- | --- | --- | --- | --- | --- |
| 0 | 0.233333 | 0.234667 | 0.252 | 0.252 | 0.23 | 0.233 |
| 2 | 0.328 | 0.328 | 0.334667 | 0.334667 | 0.328 | 0.328 |
| 4 | 0.7 | 0.703333 | 0.722667 | 0.722667 | 0.706667 | 0.693333 |
| 6 | 0.712 | 0.749 | 0.826 | 0.826 | 0.819333 | 0.712 |
| 9 | 2.456 | 2 | 2.11467 | 2.11467 | 1.88267 | 2.02867 |
| 12 | 4.548 | 4.21933 | 3.936 | 3.936 | 3.49467 | 2.93067 |
| 15 | 5.6 | 5.2 | 5.03333 | 5.03333 | 4.8 | 3.09133 |
| 18 | 6.3 | 6.06667 | 5.96667 | 5.96667 | 5.68333 | 4.27667 |
| 21 | 7.5 | 7.30667 | 6.7 | 6.7 | 6.81667 | 4.55667 |
| 24 | 8.13 | 8.09 | 7.88333 | 7.88333 | 7.58 | 4.74333 |

**Supplemantary Figure 2:** Concentrations (1-400 mg/L) applied to determine chronic toxicity doses (A) and acute toxicity doses (B) of DM for yeast.

**Supplemantary Figure 3:** Microscopic pictures of yeast cells treated with DLM at concentrations of 10 mg/L (A), 20 mg/L (B), 30 mg/L (C), 40 mg/L (D), No DM treatment (E).

**Supplemantary Figure 4:** PLS-DA model fittings of metabolomics data of yeast treated with or without DLM

**Supplemantary Figure 5:** DisGeNET disease enrichment analysis in MetaScape based on human homologs of yeast proteins.

**Supplemantary Table 1.** The list of primers used in (qRT-PCR) analysis of deltamethrin (un)treated yeast strains

| Primers Name | Directions | Sequence |
| --- | --- | --- |
| IRE1_F | 5`-3` | TTGAAGACACCCTCCAACGG |
| IRE1_R | 5`-3` | TCGAACCTCTCCTGGTTTGC |
| ACT1_F | 5`-3` | GCCTTCTACGTTTCCATCCA |
| ACT1_R | 5`-3` | GGCCAAATCGATTCTCAAAA |
| PUT4_F | 5`-3` | TACGATATGAACAGCCCCGC |
| PUT4_R | 5`-3` | TGGACTTGGAGAAATCGCCC |
| AGP1_F | 5`-3` | TCCAATCCTTGTGCCAGACC |
| AGP1_R | 5`-3` | ATTGCCCAATTTTGGGTCGC |
| FRT2_F | 5`-3` | GATGGCAAGCCTCTCACAGT |
| FRT2_R | 5`-3` | CAATCGCTGAACATTGCGGT |
| FRE3_F | 5`-3` | GAAAAACGTCAGCGGCAACT |
| FRE3_R | 5`-3` | CACATCAGCTCCGGCTTGTA |
| SIT1_F | 5`-3` | TGAAGAGGTTGTCGTTCCCG |
| SIT1_R | 5`-3` | GGGCGGTTGTACATTTCTGC |

**Supplemantary Table 2.** The ratio of dead yeast cells to alive yeast cells treated with DLM doses ranging from 1-40 mg/L

| Treatment deltamethrin doses (mg/L) | Inhibition rate (included dead cells) after treatment (%) | Inhibition rate (not-included dead cells) after treatment (%) | Dead cells rate after treatment (%) |
| --- | --- | --- | --- |
| 1 | 0 (Cell count of treated group outnumbered that of non-treated group) | 0 (Cell count of treated group outnumbered that of non-treated group) | ND* |
| 10 | ND* | ND* | ND* |
| 20 | 3.6±0.4 | 5.2±1.3 | 1.7±0.2 |
| 30 | 9.03±1.2 | 9.4±1.8 | 3.1±0.9 |
| 40 | 14.7±4.5 | 15.55±6.8 | 12.09±2.8 |

*: indicates not detected dead cells after the treatment

**Supplemantary Table 3.** The statistical results of RT-qPCR analysis of six genes including PUT4, FRT2, IRE1, AGP1, SIT1, and FRE3 (*P<0.05, **P< 0.01, ***P<0.001)

| Genes | P value of No treatment vs 30 mg/L DLM treatment | P value of No treatment vs 10 mg/L DLM treatment | P value of 10 mg/L DLM treatment vs 30 mg/L DLM treatment |
| --- | --- | --- | --- |
| PUT4 | 0.2035 | 0.002092** | 0.01386* |
| FRT2 | 0.9982 | 0.04177* | 0.04479* |
| IRE1 | 0.7943 | 0.04143* | 0.04243* |
| AGP1 | <0.0001*** | 0.0004297*** | 0.0009361*** |
| SIT1 | 0.5651 | 0.0007470*** | 0.001719** |
| FRE3 | 0.5907 | 0.001627** | 0.003983** |

**Supplemantary Table 4.** VIP scores of 12 metabolites based on the model constructed in PLS-DA

| Metabolites | VIP scores |
| --- | --- |
| Threonine | 1.2498629 |
| Norleucine | 1.2494483 |
| Phenylalanine | 1.2489553 |
| Serine | 1.2337831 |
| Isoleucine | 1.2210621 |
| Alanine | 1.2158088 |
| Proline | 1.2149753 |
| Valine | 1.2139941 |
| Octadecanoic acid | 1.1970148 |
| Glycine | 1.1736424 |
| phosphoric acid | 1.1019072 |
| Trehalose | 1.0890397 |
